# Coarse and fine root plants affect pore size distributions differently

**DOI:** 10.1007/s11104-014-2079-8

**Published:** 2014-03-14

**Authors:** G. Bodner, D. Leitner, H.-P. Kaul

**Affiliations:** 1 Department of Crop Sciences, Division of Agronomy, University of Natural Resources and Life Sciences, Konrad-Lorenz-Strasse 24, 3430 Tulln, Austria; 2 Computational Science Center, University of Vienna, Oskar Morgenstern-Platz 1, 1090 Vienna, Austria

**Keywords:** Pore size distribution, Cover crops, Root system, Conceptual model, Soil structure, Pore evolution

## Abstract

**Aims:**

Small scale root-pore interactions require validation of their impact on effective hydraulic processes at the field scale. Our objective was to develop an interpretative framework linking root effects on macroscopic pore parameters with knowledge at the rhizosphere scale.

**Methods:**

A field experiment with twelve species from different families was conducted. Parameters of Kosugi’s pore size distribution (PSD) model were determined inversely from tension infiltrometer data. Measured root traits were related to pore variables by regression analysis. A pore evolution model was used to analyze if observed pore dynamics followed a diffusion like process.

**Results:**

Roots essentially conditioned soil properties at the field scale. Rooting densities higher than 0.5 % of pore space stabilized soil structure against pore loss. Coarse root systems increased macroporosity by 30 %. Species with dense fine root systems induced heterogenization of the pore space and higher micropore volume. We suggested particle re-orientation and aggregate coalescence as main underlying processes. The diffusion type pore evolution model could only partially capture the observed PSD dynamics.

**Conclusions:**

Root systems differing in axes morphology induced distinctive pore dynamics. Scaling between these effective hydraulic impacts and processes at the root-pore interface is essential for plant based management of soil structure.

## Introduction

Soil hydraulic properties are the common result of particle size distribution (texture) and aggregation (structure). Soil structure is fundamental for the shape of water retention and hydraulic conductivity in the saturated and near-saturated range (Cresswell et al. 1992). Among the various driving factors of soil structural porosity, vegetation plays a dominant role. Roots are a key element in plant related effects on soil structure and soil hydrology (Gregory 2006; Pierret and Moran 2011; Bengough 2012; Logsdon 2013). The classical hierarchy model of structured soil (Tisdall and Oades 1982) highlights the direct and indirect role of plant roots as binding agents at various levels. Following the aggregate hierarchy model, Elliot and Coleman (1988) defined four functionally related categories for the pore space, i.e. large macropores (root channels, earthworm holes, shrinkage cracks), inter-macroaggregate, inter-microaggregate and intra-microaggregate pores.

Several pathways of root influence on soil hydraulic properties have been proposed. Direct root influence was related to temporal pore clogging due to roots growing into pre-existing pores (e.g. Gish and Jury 1983; Morgan et al. 1995). Scanlan (2009) suggested that root in-growth results in the division of larger into smaller pores. After root decay, bio-macropores and root-induced micropores are formed (Cresswell and Kirkegaard 1995; Mitchell et al. 1995; Wuest 2001; Horn and Smucker 2005; Ghestem et al. 2011). These pores with high connectivity (Pagliai and De Nobili 1993; Whalley et al. 2005) facilitate water transport through the soil (Gish and Jury 1983; Murphy et al. 1993; Suwardji and Eberbach 1998). Thus biologically induced pores not only differ in size but also geometry, pointing to the need to go beyond traditional capillary bundle models to properly capture root-pore effects (Hunt et al., 2013).

Mechanical effects of growing roots are related to axial and radial pressures exerted during soil penetration. They can cause enlargement of existing pores and densification of adjacent rhizosphere soil (Dexter 1987; Archer et al. 2002; Kirby and Bengough 2002; Whalley et al. 2004). Crack formation and micro-fissuring by enhanced wetting-drying were also proposed as relevant mechanisms of root induced pore formation (e.g. Dexter 1987; Mitchell et al. 1995; Young 1998; Whalley et al. 2005).

Biochemical effects of roots on hydraulic properties, both directly on the pore channels and indirectly via aggregation, have been described in relation to (1) structure formation and stabilization by root organic matter and exudates (Czarnes et al. 2000; Lado et al. 2004), (2) water repellence of root-derived organic compounds (Hallett et al. 2003) and (3) organic matter effects on water holding capacity (Hudson 1994; Dexter 2004). Carminati and Vetterlein (2013) showed that mucilage effects on rhizosphere hydraulic properties varied with root age and soil moisture.

The importance of root influence on the soil pore system is controlled by both soil and root characteristics. Based on CT imaging, Luo et al. (2010) demonstrated a significant interaction effect between land use (pasture vs. crop differing in rooting density and organic matter) and soil type (sand vs. silt loam) on macropore properties. Scanlan (2009) did not find a root effect on soil hydraulic properties in a column experiment using a sandy substrate. We assume that changes of pore properties are dependent on both the relation between root volume and pore volume (Bengough 2012) and the extent of existing growth paths used by roots upon soil penetration (Feeney et al. 2006).

Yunusa and Newton (2003) reported differences among species in their effects on soil hydraulic properties. Perennials and woody plants substantially changed flow behavior while annual crops had hardly any influence. Among annual plants they suggested root diameter as main trait for effectively priming the soil pore space. Higher strength of coarse roots allows more effective shift of soil particles and lower tendency of root buckling under mechanical stress (Clark et al. 2003). Using a pore network model, Holtham et al. (2007) showed different root-induced soil structuring between white clover and ryegrass with enhanced macroporosity under the coarse rooted legume.

In spite of increasing knowledge on root-soil interactions, targeted management of soil structure by roots (“bio-tillering”) is still at its infancy. Yunusa and Newton (2003) presented the concept of primer-plants, i.e. plants without a direct economic benefit, but effective in conditioning the soil for cash crops and in conserving environmental resources. Cover crops correspond to this type of plants. Currently they are used in agro-environmental programs to minimize nitrate leaching and reduce soil erosion. Several authors observed cover crop effects on soil structural properties such as aggregate size and stability (Liu et al. 2005) as well as hydraulic processes such as water infiltration (Carof et al. 2007; Bodner et al. 2008).

While there is significant advance in understanding root-soil processes via modern imaging methods (e.g. Feeney et al. 2006; Moradi et al. 2011), we identified two shortcomings: (i) most studies were based on one or few species only, thereby lacking variability in root traits to properly quantify the extent of root influences on soil properties; (ii) small scale rhizosphere processes were rarely evaluated for their impact on a representative elementary volume (REV) relevant for field scale hydraulic processes and under conditions where they co-exists with other structure forming processes. This however is a pre-condition to infer the role of roots for management of soil physical quality.

The objectives of our study therefore were (i) to identify root induced changes of field measured macroscopic pore parameters under different autumn grown cover crop species, (ii) to analyse if different root systems distinctively modify soil pore properties, and (iii) to provide a conceptual framework that links macroscopic root effects with relevant rhizosphere processes of soil structure formation.

## Material and methods

### Experimental site

Measurement data were obtained from a field experiment at the Experimental Station Groß Enzersdorf of the University of Natural Resources and Life Sciences, located in Lower Austria (48°14′N, 16°35′E, 156 m asl). Climatically the site is characterized by sub-humid conditions (pannonic) with an average annual precipitation of 525 mm, a mean annual temperature of 9.8 °C, and a mean relative humidity of 75 %. The soil at the site is classified as Chernozem according to the WRB (IUSS 2006). Basic soil properties are given in Table 1.

**Table 1:** Soil properties of the experimental field

Horizon	Depth cm	Sand kg kg^−1^	Silt kg kg^−1^	Clay kg kg^−1^	Texture USDA	C_org_ kg kg^−1^	Field capacity cm^3^ cm^−3^	Wilting point cm^3^ cm^−3^
A	0–40	0.19	0.56	0.24	SiL	0.025	0.32	0.15
AC	40–55	0.23	0.54	0.23	SiL	0.015	0.27	0.10
C	> 55	0.22	0.62	0.16	SiL	0.008	0.25	0.07

The field experiment comprised twelve species which are commonly used as cover crops. The species belonged to different plant families (Table 2). Based on available description by Kutschera et al. (2009), distinct root system characteristics of the species could be expected.

**Table 2:** Investigated cover crop species and their respective plant families

Species	Family
*Vicia sativa* L.	Fabaceae
*Lathyrus sativus* L.	Fabaceae
*Trifolium alexandrinum* L.	Fabaceae
*Melilotus officinalis* L.	Fabaceae
*Sinapis alba* L.	Brassicaceae
*Raphanus sativus var. oleiformis* L.	Brassicaceae
*Phacelia tanacetifolia* Benth.	Boraginaceae
*Linum usitatissimum* L.	Linaceae
*Fagopyrum esculentum* Moench.	Polygonaceae
*Secale cereale* L.	Poaceae
Mixture 1 (*Secale cereale* L.*, Trifolium incarnatum* L*., Vicia villosa* Roth.)	–
Mixture 2 (*Phacelia tanacetifolium* Benth.*, Sinapis alba* L., *Vicia sativa* L.)	–

The experimental design was a randomized complete block design (RCBD) with three replicates. Plot size was 4.5 m^2^ (1.5 m × 3 m). Seeding date was on 28th July 2011. Previously to cover crop seeding, the field was cropped with winter wheat which was harvested on 10th July and thereafter stubble tilled with a chisel plow to a soil depth of 10 cm. Rainfall during the cover crop growing period (28th July to first frost on 22nd November) was 140.2 mm compared to 180 mm long-term average. While August had high precipitation, September and November were clearly drier compared to long-term averages. Mean monthly temperature during the growing period decreased from 20.9 °C in August to 2.9 °C in November, with a base temperature for plant growth above 5 °C until 10th of November.

### Root sampling and analysis

Root samples were taken by the ‘soil-core’ method (Böhm 1979). Soil cores (250 cm^3^) were extracted at the end of the cover crop vegetation period when most species had reached their maximum growth before winter. Samples were taken from surface-near soil (2–7 cm soil depth). Root sampling depth corresponded to the visually observed depth of the infiltration front during tension infiltrometer measurements (*cf.* 2.3). Hydraulic properties and root traits were thus representative for the surface near layer with highest rooting densities and most structural dynamics in the soil. Three subsamples per plot were taken at the same position of infiltration measurements, giving a total number of 108 sampling points.

After field sampling, roots were washed free of soil in the laboratory over a set of sieves (2 mm and 0.5 mm mesh screen). An extra sieve of 0.2 mm was placed under the 0.5 mm sieve in order to avoid fine roots loss. Following removal of soil, roots were separated from dead roots of previous crop and organic debris with tweezers based in differences in color and flexibility. Roots were then stained with methylene-blue and morphological parameters were determined by image analysis using WinRhizo 4.1 (Regent Instruments, Quebec). Following measurement of root morphological parameters, root dry mass was determined after drying to constant weight at 60 °C.

### Tension infiltrometer measurements

Infiltration experiments were conducted between 25th October and 15th November 2011. The measurements were performed using a tension infiltrometer (Soil Measurement Systems Inc., Tucson, AZ) with a 20 cm diameter disc. A total amount of 108 measurements (12 species × three replicates × three subsample) were taken at the soil surface after carefully removing mulch and any above-ground plant material. Additionally a non-planted control was included.

A nylon mesh to avoid macropore clogging and a fine layer of quartz sand (diameter: 0.08–0.2 mm) to ensure good hydraulic contact were placed between the disc and the soil. The supply pressure heads were −15, −10, −5, −1 and 0 cm. The first two pressure heads were maintained for approximately 40–60 min, and the higher pressure heads were applied for about 10–15 min. Preliminary tests found these durations to be sufficient to achieve steady-state infiltration. The water level in the supply tube was observed visually in intervals of 15 s during the first 5 min after application of a supply pressure, and in increasing intervals of 2–10 min afterwards. Before each infiltration measurement, soil samples were taken with steel cores (250 cm^3^) in the vicinity of the measurement location to obtain the initial water content, bulk density and total porosity. Immediately after each infiltration measurement, another core sample was collected directly below the infiltration disc to quantify the final water content.

### Inverse estimation of soil hydraulic properties

The inverse analysis of tension infiltrometer data to estimate soil hydraulic properties requires a numerical solution of the Richards’ equation for radially symmetric Darcian flow. We followed the procedure presented by Šimůnek et al. (1998). Soil water retention and hydraulic conductivity were described by the model of Kosugi (1996) which is based on a lognormal pore-size distribution (PSD). Soil water retention S_e_(h) is given by

1 $$ {S}_e(h)=0.5 erfc\left(\frac{ \log \left(\frac{h}{h_{m, Kosugi}}\right)}{\sqrt{2{\sigma}_{Kosugi}}}\right) $$

where S_e_ (−) is the effective saturation corresponding to $\frac{\theta -{\theta}_r}{\theta_s-{\theta}_r}$ with θ_r_ (cm^3^ cm^−3^) being residual water content and θ_s_ (cm^3^ cm^−3^) saturation water content. *Erfc* is the complementary error function, h_m,Kosugi_ (cm) the median pressure head and *σ*_Kosugi_ (−) the standard deviation of the log-transformed pressure head.

Hydraulic conductivity K(h) can be written as

2 $$ K(h)=\left\{\begin{array}{l}{K}_s{S_e}^l{\left\{\frac{1}{2} erfc\left[\frac{ \ln \left(\frac{h}{h_{m, Kosugi}}\right)}{\sqrt{2{\sigma}_{Kosugi}}}+\frac{\sigma_{Kosugi}}{\sqrt{2}}\right]\right\}}^2\kern2em \left(h<0\right)\\ {}\kern4em {K}_s\kern12em \left(h\ge 0\right)\end{array}\right. $$

where K_s_ (cm s^−1^) is saturated hydraulic conductivity and *l* (−) is a tortuosity factor.

Parameter estimation was done by minimizing the objective function between observed and predicted cumulative infiltration and final water content following Šimůnek and Van Genuchten (1996) using the program HYDRUS 2D/3D (Šimůnek et al. 2006) which applies a Levenberg-Marquardt nonlinear minimization algorithm. Initial parameter estimates were derived from the texture based pedotransfer function Rosetta (Schaap et al. 2001). To reduce the number of unknown variables, θ_r_ and *l* were fixed to 0.067 cm^3^ cm^−3^ and 0.5 respectively, as predicted by Rosetta. K_s_ values were used from direct Wooding analysis of infiltration data, and θ_s_ was taken equal total porosity obtained from sample cylinders. The remaining parameters, *σ*_Kosugi_ and h_m,Kosugi_, were then estimated inversely.

### Simulation of pore evolution

Or et al. (2000) presented a convection–dispersion type model for pore evolution. We applied their model to analyze if changes in PSD by differently rooted species can be described by the physics underlying this approach. Pore size distribution *f* is the first derivative of the retention curve and can be written as

3 $$ f(r)=\frac{\theta_s-{\theta}_r}{\sigma\;r\sqrt{2\pi }} \exp \left\{-\frac{{\left[ \ln \left(r/{r}_m\right)\right]}^{{}^2}}{2{\sigma}^2}\right\} $$

where *r* (µm) is the pore radius, *r*_m,Kosugi_ (µm) is the median pore radius, and σ_Kosugi_ (−) is its standard deviation. The median pore radius can be calculated from the median pressure head h_m,Kosugi_ using the Young-Laplace equation.

According to the pore evolution model changes of the PSD can be described by

4 $$ \frac{\partial f}{\partial t}=\frac{\partial }{\partial r}\left(D\left(r,t\right)\frac{\partial f}{\partial r}\right)-\frac{\partial }{\partial r}\left(V\left(r,t\right)f\right)-M(t)f $$

where *t* is time, *V* (µm s^−1^) is a drift term, *D* (µm^2^ s^−1^) a dispersion term and *M* (s^−1^) a degradation term. The drift and dispersion terms quantify changes of *r*_*m,Kosugi*_ and σ_Kosugi_ respectively. *M* represents a sink term for changes in total porosity. Dispersion is related to drift by a constant dispersivity λ (µm).

The model was parameterized using an analytical solution of Eq. 4 developed by Leij et al. (2002). The governing parameters in this solution are the cumulative drift *T*, equal the integral of *V*, and dispersivity λ, which were optimized from the measured PSDs. *M* was set equal the reduction in total porosity. While other authors limited degradation to the macropore range (e.g. Schwärzel et al. 2011), due to the lack of proper data, we did not attribute degradation to any distinct pore range. All calculations of pore evolution were done with Matlab Version 8 R2012b.

### Statistical evaluation

Statistical data evaluation was performed by analysis of variance with the procedure PROC MIXED in the software SAS 9.2. This procedure is based on restricted maximum likelihood estimates of the variance components and provides Wald-type F-statistics using GLSE (generalized least squares). In case of significant effects at *p* ≤ 0.05 in the analysis of variance, comparison of means was performed using a two-sided *t*-test. In order to test hypotheses on differences among groups of species with similar root and soil parameters we used linear contrasts which were obtained by the CONTRAST statement in PROC MIXED.

For root system characterization we also applied a multivariate approach based on principal component analysis (PROC FACTOR) and clustering (PROC CLUSTER). This method was suggested for functional root system classification and is described in detail by Bodner et al. (2013a).

Regression analysis was used to find significant root predictor variable for PSD parameters. For this purpose we used the SAS procedure PROC REG with the RSQUARE selection method.

## Results

### Root system characteristics

Root systems of twelve cover crop species were characterized by morphological traits and parameters of a lognormal root volume distribution model suggested by Scanlan and Hinz (2010) to capture root volume allocation to different root radius classes (Table 3).

**Table 3:** Traits of root morphology and volume allocation of twelve cover crop species from different plant families. Values characterize the surface near (2–7 cm) rooting pattern of the species.

	RLD^a^ (cm cm^−3^)	RVD (cm^3^ cm^−3^)	RD (mm)	SRL (m g^−1^)	r_m,root_ (mm)	ς_root_ (−)
*Species*						
* V. sativa*	3.57def	0.0053bc	0.47a	73.3gh	0.31	0.65cde
* L. sativus*	3.04def	0.0058ab	0.49a	80.5fgh	0.34	0.62de
* T. alexandrinum*	2.70ef	0.0038 cd	0.39bc	118.3d-h	0.35	0.76a-e
* M. officinalis*	1.88f	0.0026de	0.41b	57.1 h	0.33	0.77a-d
* Mixture 1*	3.04def	0.0075a	0.36 cd	130.2c-g	0.32	0.75b-e
* S. alba*	4.05cde	0.0027de	0.28e	194.5abc	0.33	0.90ab
* R. sativus*	4.87bcd	0.0033de	0.30e	161.9a-e	0.32	0.99a
* Mixture 2*	6.64b	0.0033de	0.35 cd	97.2e-h	0.37	0.85a-d
* P. tanacetifolia*	5.71bc	0.0038 cd	0.29e	184.5a-d	0.30	0.96ab
* L. usitatissimum*	11.02a	0.0068ab	0.31de	212.4a	0.21	0.86abc
* F. esculentum*	1.79f	0.0018e	0.34cde	200.4ab	0.41	0.82a-d
* S. cereale*	2.41ef	0.0020de	0.33cde	142.5b-f	0.28	0.53e
Species	***	***	***	***	ns.	**
LSD^b^	2.03	0.0019	0.05	66.7	0.17	0.23
CV%^c^	64.8	50.5	19.7	44.6	33.0	22.2

Means followed by the same letter within a column are not significantly different at *p* ≤ 0.05; ns. not significant; ** significant at *p* ≤ 0.01; *** significant at *p* ≤ 0.001

^a^ *RLD* Root length density, *RVD* Root volume density, *RD* Root diameter, *SRL* Specific root length, *r*_*m,root*_ Median root radius, *ς*_*root*_ Standard deviation of lognormal root volume distribution

^b^ *LSD* Least significant difference

^c^ *CV*%, Coefficient of variation

All parameters except r_m,root_ of the lognormal distribution showed significant differences between species. *F. esculentum* and *S. cereale* had lowest rooting density. In case of *S. cereale* this was linked to low aboveground growth (982.5 kg ha^−1^). *F. esculentum* however had higher aboveground biomass (2033.3 kg ha^−1^) and thus a comparatively low dry-matter allocation to the root system. Most species showed an intermediate aboveground growth with an average dry-matter of 2,051 kg ha^−1^, which is within the range of values reported for cover crops in this region.

Non-legume cover crops showed higher rooting density (RLD, RVD) and more biomass allocation to fine roots (high SRL), while legume species had higher RD and lower SRL. The median radius of root volume distribution over radius did not differ significantly between species, but its standard deviation was significantly larger for the non-legume species.

**Fig. 1:**
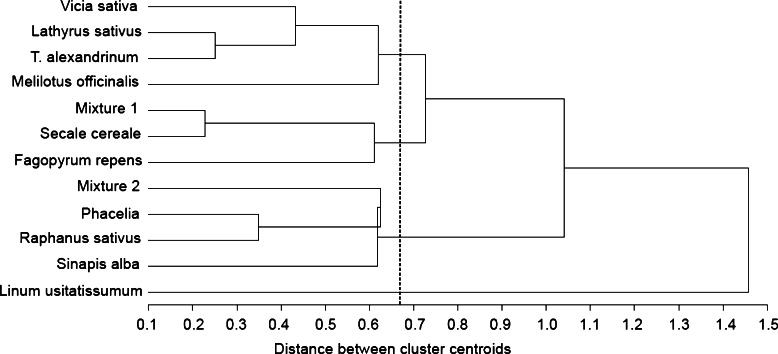
Species sharing similar rooting types determined from cluster analysis and using morphologically based principal components as classification variable

Following Bodner et al. (2013a) we used principal component and cluster analysis for a multivariate characterization of similarities among root systems integrating all single morphological descriptors. Results are shown in Fig. 1.

Four groups were suggested by the cubic clustering criterion. The dendrogram separated legumes at one end, while *L. usitatissmum* formed a separate group at the opposite side. The density dominated rooting types (Brassicaceae, *P. tanacetifolia*, Mixture 2) were in a common group, while *S. cereale*, *F. esculentum* and the legume-grass mixture 1 were between the diameter and density dominated rooting types. Fabaceae shared a high diameter/low density rooting type (similar score on principal component 2 containing the common effects of RD, SRL and ς_root_). Brassica species were in a joint cluster of fine root dominated dense rooting type (similar score on principal component 1 containing effects of RLD, RVD and r_m,root_). However this cluster was not specific to Brassicaceae, but contained species from different families.

### Soil pore size distribution

The average values of soil PSD parameters obtained by inverse optimization (h_m,Kosugi_ 41.1 cm, σ_Kosugi_ 2.14) were between those of sandy loams (h_m,Kosugi_ 27.4 cm, σ_Kosugi_ 1.26) and silty loams (h_m,Kosugi_ 325.9 cm, σ_Kosugi_ 2.30) indicated by Šimůnek (2006). The peak in volumetric PSD corresponds to the pore radius with highest frequency. The dominant pore class in the PSDs was ultramicropores (r < 2.5 µm; SSSA 2013) with highest frequencies between 0.05 and 1.7 µm for *P. tanacetifolia* and *T. alexandrinum* respectively (Figure not shown). The less frequent coarser pore classes however contribute essentially to total pore volume. This is expressed by r_m,Kosugi_ which was two to three orders of magnitude higher than the most frequent pore radius (between 33.7 µm and 91.3 µm for *P. tanacetifolia* and *L. sativus* respectively). Table 4 gives the Kosugi parameters for each cover crop species.

**Table 4:** PSD parameters of soil under different cover crop species. Species with similar overall PSD are grouped together

	Species	θ_s_ cm^3^ cm^−3^	r_m,Kosugi_ μm	ς_Kosugi_ _−_
Group 1 High porosity – high median radius	*L. sativus*	0.471ab	91.3a	2.19abc
	*Mixture 1*	0.478a	79.1abc	2.28abc
	*M. officinalis* ^a^	0.442bcd	85.4ab	2.15bc
	*Mean*	0.463A	85.3A	2.21A
Group 2 High pore radius range	*P. tanacetifolia*	0.446abc	33.7d	2.46a
	*L. usitatissimum*	0.469ab	52.8bcd	2.31ab
	*R. sativus*	0.457abc	52.7bcd	2.40ab
	*V. sativa*	0.463ab	65.8abcd	2.34ab
	*Mean*	0.459A	51.3B	2.38B
Group 3 Low pore radius range	*S. alba*	0.456abc	50.8bcd	1.89c
	*Mixture 2*	0.465ab	81.7abc	1.90c
	*T. alexandrinum* ^a^	0.436bcd	89.0ab	1.84c
	*Mean*	0.452A	73.8A	1.88C
Group 4 Low porosity – low median radius	*S. cereale*	0.431 cd	47.0de	2.25abc
	*F. esculentum*	0.413d	46.0 cd	1.95bc
	*Mean*	0.422B	46.5B	2.10 AC

Common lower-case letters at the respective parameters indicate non-significant differences at *p* ≤ 0.05. Group means are compared by linear contrasts. Significant differences in parameter means at *p* ≤ 0.05 between groups are indicated by upper-case letters

^a^Intermediate species not clearly related to a single groups

Using linear contrasts, four groups with similar PSD were obtained which had no significant within-group differences and a distinct hydraulic behavior towards a contrasting group (i.e. significant between-group difference in at least one parameter). The parameter averages for these four groups are also given in Table 4

Groups 1 and 4 differed in θ_s_ and r_m,Kosugi_. Species in group 1 had high values in both parameters except *M. officinalis* with low θ_s_. Those in group 4 were low in both parameters; particularly *F. esculentum* had low values for all PSD parameters. Group 2 and 3 differed in ς_Kosugi_ while having an intermediate porosity and a range of different r_m,Kosugi_ values. Group 2 contained species with high σ_Kosugi_, while species in group 3 had a narrow PSD.

*M. officinalis* and *T. alexandrinum* could not be attributed clearly to a single group. Both had a high r_m,Kosugi_ similar to species in group 1, but lower θ_s_. *M. officinalis* had an intermediate s_Kosugi_, while in this parameter *T. alexandrinum* corresponded clearly to species with narrow pore range in group 3.

For comparison, we mention the average Kosugi parameters of unplanted control plots which were θ_s_ = 0.418 cm^3^ cm^−3^, r_m,Kosugi_ = 48.8 µm, and ς_Kosugi_ = 2.12 and similar to those of species in group 4.

### Root influences on PSD parameters

**Fig. 2:**
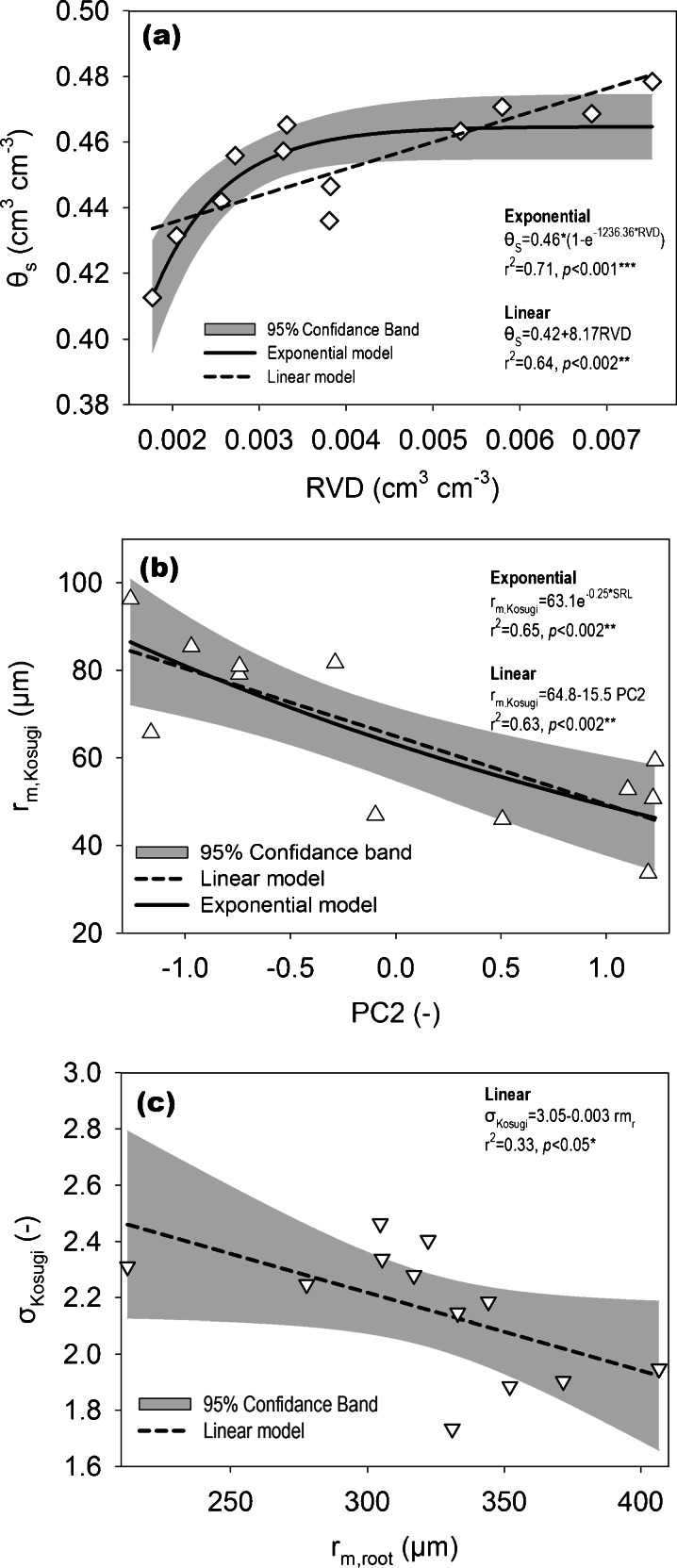
Relations between macroscopic PSD parameters and best root predictor variables. Non-linear functions are shown in case of providing better fit compared to linear regressions

Relations between field measured root traits and pore characteristics were determined by regression analysis. Figures 2a-c show the best root predictor variables for the respective macroscopic pore parameters of the Kosugi model.

θ_s_ had a strong positive relation to RVD (Fig. 2a). The most appropriate functional form for this relation was a curve rising exponentially to a maximum of θ_s_ = 0.46 cm^3^ cm^−3^ with RVD > 0.004 cm^3^ cm^−3^. From this relation it is clear that even small increments beyond a minimum rooting density had a strong effect on soil porosity while highly dense root systems did not further increase the pore volume.

Also r_m,Kosugi_ showed a clear significant relation to rooting traits (Fig. 2b). The highest R^2^ (0.65) was achieved using principal component 2 (PC2) containing the common effects of SRL, RD and ς_root_. All single parameters showed a significant relation to r_m,Kosugi_ for their own, which however had a slightly lower R^2^ than the composite variable. An exponentially decaying function obtained a slightly better fit compared to a linear relation. We also mention here that soil moisture strengthened this root effect. An R^2^ of 0.76 of a bivariate linear regression with root PC2 and soil moisture as predictor variables highlighted this common effect.

For ς_Kosugi_ there was only a weak, but still significant, linear relation to the median radius of the lognormal root volume distribution (Fig. 2c). Root systems with volume allocated to finer axes (low r_m,root_) tended to induce a slightly higher soil pore radius standard deviation.

### Conceptual model of root induced modification of PSD

**Fig. 3:**
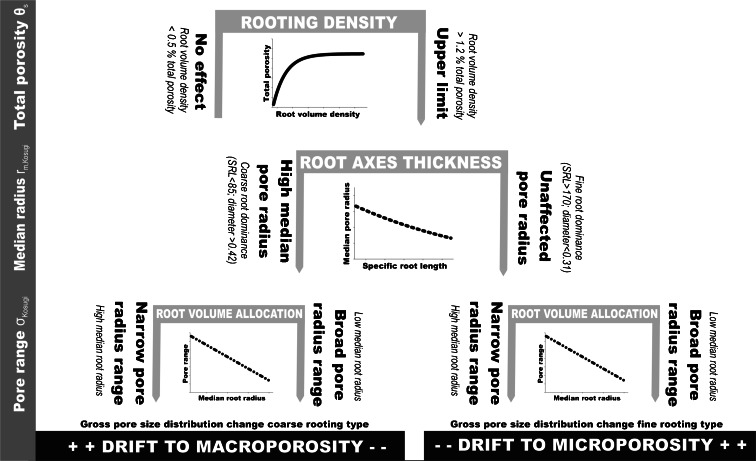
Conceptual model of root influences on the parameters of Kosugi’s macroscopic model of soil PSD. Beyond a minimum rooting density (effect vs. no effect threshold) two distinct pathways for coarse vs. fine axes dominated root system and the resulting changes in pore size distribution are shown. (Graphs of root-pore relations are schematic representations of the regressions shown in Fig. 2)

Figure 3 shows a conceptual model of root influences on PSD built from the relations between root traits and pore parameters (*cf.* Figure 2a-c) as well as the grouping of species based on similarities in root and pore parameters (Table 4).

The exponential relation between RVD and θ_s_ (*cf.* Figure 2a) indicated an upper and lower limit for root effects on soil porosity. From our data the lower limit, where roots did not substantially condition the soil pore space (*cf.* Table 4, group 4), was in the range of 0.5 % of pore volume occupied by roots. The upper limit was achieved at a RVD occupying more than 1.2 % of total soil porosity.

**Fig. 4:**
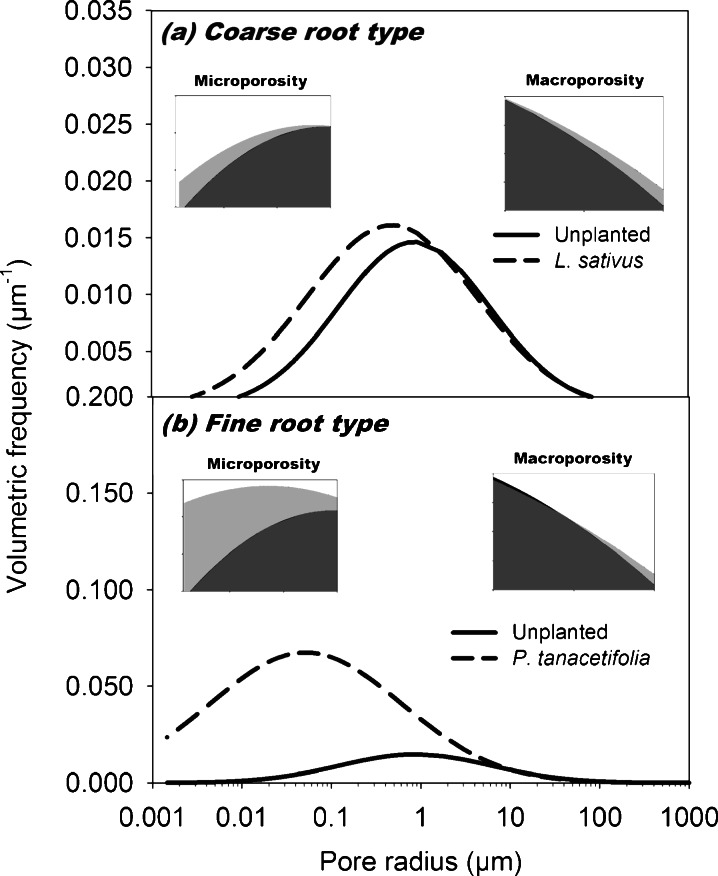
Example of changes in PSD between unplanted soil and soil influenced by roots of species with (**a**) coarse and (**b**) fine root axes morphology. Small figures at the top highlight differentiation in micropore and macropore range (log-log scale; *light grey* shows range with higher pore frequency of planted vs. un-planted, *black* shows range of lower pore frequency, *dark gray* shows overlapping pore frequency)

In case of a sufficiently dense root system to modify soil porosity, there was a fundamental difference between species dominated by coarse and fine root axes (*cf.* Figure 2b). While coarse root systems induced a drift towards increased macroporosity (high r_m,Kosugi_; *cf.* Table 4, group 1 and legumes in group 3), fine axes did not change significantly r_m,Kosugi_ compared to non-planted plots and treatments with negligible root effects (*F. esculentum, S. cereale*). This was reflected in significant contrasts between legumes and non-legumes in parameters capturing root axes thickness and correspondingly in their soil r_m,Kosugi_. Thresholds of the main root traits involved in this effect were estimated from these contrasts. They were in the range of SRL values < 85 m g^−1^ and root diameters > 0.42 for species enhancing r_m,Kosugi_. Low r_m,Kosugi_ was found for species with an average SRL > 170 m g^−1^ and root diameter values < 0.31 mm. This distinct effect of contrasting root axes morphology on PSD is exemplified in Fig. 4 for two characteristic species of each type (*P. tanacetifolia* and *L. sativus*). PSD of an unplanted control is given as reference state.

*L. sativus* resulted in a 43.9 % increase of macroporosity (> 37.5 µm) due to high r_m,Kosugi_, while micropore volume decreased by 17.5 % compared to the reference state. *P. tanacetifolia* on the contrary substantially increased the frequency of fine pores, resulting in a micropore volume (< 15 µm) 45.3 % higher than the reference state. Macropores on the contrary were reduced by 2.1 %. This however was not only related to the low r_m,Kosugi_ (33.7 µm) of this species, but mainly to a high ς_Kosugi_. Mesopores decreased in both species by 9.8 % (Table 5).

**Table 5:** Volume of different pore radius classes in soil influenced by species with coarse and fine root axes morphology exemplified by *L. sativus* and *P. tanacetifolia* respectively

	Pore volume *cm* ^*3*^ *cm* ^*−3*^		
	Unplanted *-*	Coarse root axes *L. sativus*	Fine root axes *P. tanacetifolia*
Micropores1 (r < 2.5 µm)	0.023	0.019	0.055
Micropores2 (2.5 ≤ r < 15 µm)	0.074	0.061	0.086
Mesopores (15 ≤ r < 37.5 µm)	0.061	0.055	0.055
Macropores1 (37.5 ≤ r < 500 µm)	0.149	0.178	0.131
Macropores2 (r ≥ 500 µm)	0.038	0.091	0.052

The influence of root traits on ς_Kosugi_ was less evident, as shown above (*cf.* Figure 2c). Still our data indicated a trend towards a modification of ς_Kosugi_ via r_m,root_. Non-legume species with dense fine axes dominated root systems showed stronger differentiation in ς_Kosugi_. Significant linear contrasts between species with low ς_Kosugi_ vs. high ς_Kosugi_ and their respective r_m,root_ (0.35 vs. 0.28 mm) could be found here. It should be noticed that for this group of species there was also the strongest relation of ς_Kosugi_ to soil moisture, indicating a fundamental role of capillary driven coalescence. Although the functional relation of ς_Kosugi_ with r_m,root_ was also evident in the coarse axes group, their overall differentiation was lower. The maximum distance in r_m,root_ was between *T. alexandrinum* vs. *V. sativa* with values of 0.31 vs. 0.35 mm. Thus the significant contrast in ς_Kosugi_ between *T. alexandrinum* (low ς_Kosugi_) and the other legume species was not reflected in a significant difference between their r_m,root_.

**Fig. 5:**
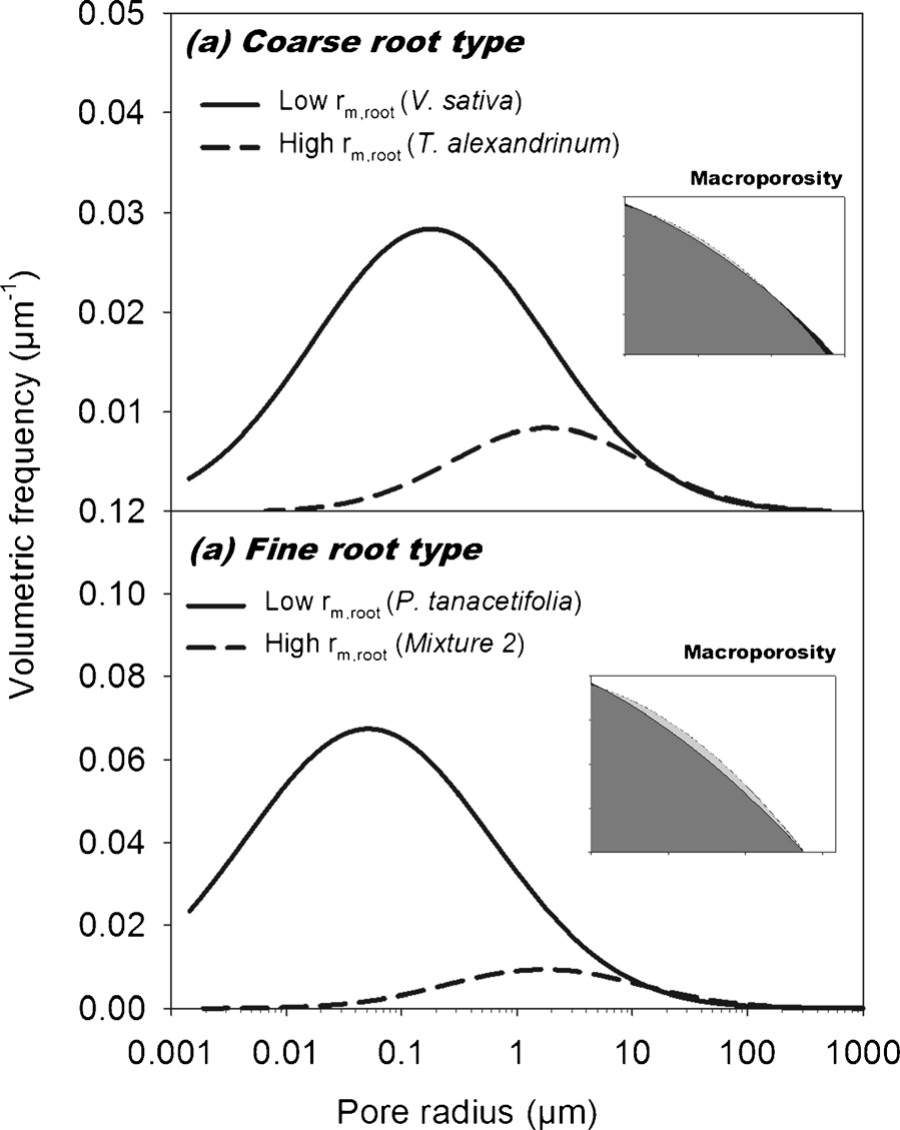
Example of changes in PSD due to different ς_Kosugi_ induced by *high* and *low* r_m,root_ for the groups with species having (**a**) coarse and (**b**) fine root axes morphology. Small figures at the *top* highlight differentiation in micropore and macropore range (log-log scale; *light grey* shows range with higher pore frequency of planted vs. un-planted, *black* shows range of lower pore frequency, *dark gray* shows overlapping pore frequency)

Examples for the effect of r_m,root_ on ς_Kosugi_ within the two groups of rooting types are shown in Fig. 5, corresponding pore volumes are give in Table 6. The higher standard deviation of pore radii induced a clear increase in micropore volume, while decreasing macropore volume particularly in the pore radius class between 37.5 and 500 µm.

**Table 6:** Volume of different pore radius classes in soil influenced by species with different median root radius within sub-groups of coarse and fine rooted species exemplified by *P. tanacetifolia* vs. *Mixture 2* and *V. sativa* vs. *T. alexandrinum*

	Pore volume *cm* ^*3*^ *cm* ^*−3*^			
	Coarse root dominated type		Fine root dominated type	
	*Low r*_*m,root*_ *V.sativa*	*High r*_*m,root*_ *T. alexandrinum*	*Low r*_*m,root*_ *P. tanacetifolia*	*High r*_*m,root*_ *Mixture 2*
Micropores1 (r < 2.5 µm)	0.032	0.010	0.055	0.013
Micropores2 (2.5 ≤ r < 15 µm)	0.072	0.052	0.086	0.061
Mesopores (15 ≤ r < 37.5 µm)	0.056	0.056	0.055	0.062
Macropores1 (37.5 ≤ r < 500 µm)	0.159	0.187	0.131	0.195
Macropores2 (r ≥ 500 µm)	0.077	0.064	0.052	0.068

Generally there was a tradeoff between macro- and microporosity (Figure not shown, R^2^ = 0.59) which was most evident (R^2^ = 0.76) when excluding the two sparsely rooted species (*F. esculentum, S. cereale*) with no overall effect on soil porosity. This underlines that – once exceeded a lower limit RVD and roots stabilizing the pore system – different rooting types induced distinctive pore evolution.

### Simulation of root induced pore dynamics

**Fig. 6:**
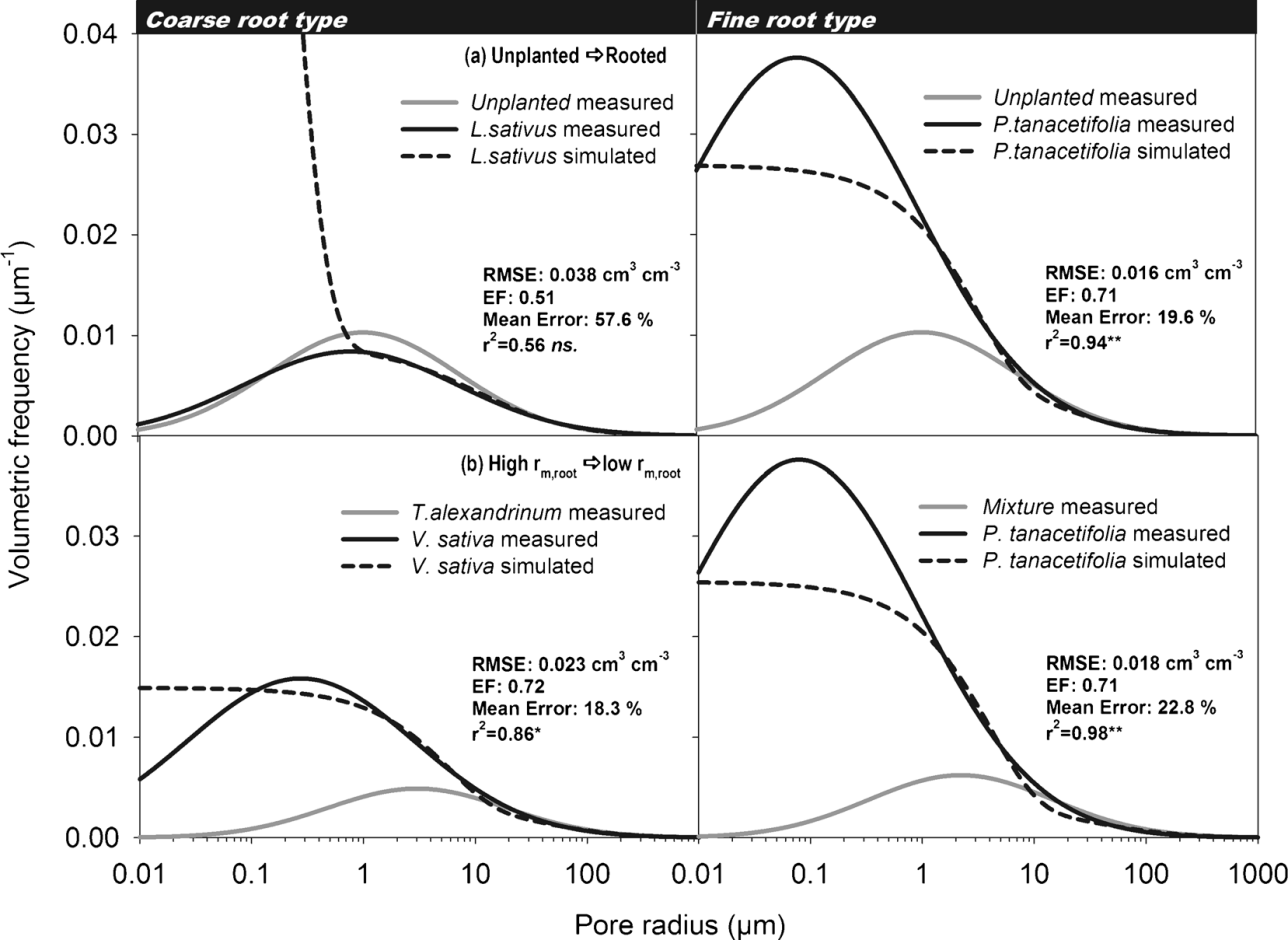
Measured and predicted PSD of soil under coarse and fine rooted species using a pore evolution model. **a** Evolution from an unplanted soil to a rooted soil, and (**b**) evolution driven by root volume allocation between a soil planted with high and low r_m,root_-species. Statistical indicators compare measured and predicted pore volume in different radius ranges given in Table 7

According to the model of Or et al. (2000) pore dynamics follow a diffusion like process. Volumetric pore frequency tends to a more even distribution with time upon shifting of the median from larger to smaller radii and simultaneously widening of the pore range (increase of ς_Kosugi_). Figure 6 shows measured and simulated PSDs according to this model for the cases of root driven pore evolution identified in Figs. 4 and 5. The corresponding pore volumes in different pore classes are given in Table 7.

**Table 7:** Measured and simulated pore volume in different radius classes for the PSDs shown in Fig. 7

	Pore volume *cm* ^*3*^ *cm* ^*−3*^						
	*P. tanacetifolia*			*L. sativus*		*V. sativa*	
	Measured	Simulated (a)	Simulated (b)	Measured	Simulated	Measured	Simulated
Micropores1 *(r < 2.5 µm)*	0.055	0.050	0.049	0.019	0.055	0.032	0.031
Micropores2 *(2.5 ≤ r < 15 µm)*	0.086	0.078	0.077	0.061	0.063	0.072	0.070
Mesopores *(15 ≤ r < 37.5 µm)*	0.055	0.050	0.041	0.055	0.057	0.056	0.046
Macropores1 *(37.5 ≤ r < 500 µm)*	0.131	0.140	0.159	0.178	0.152	0.159	0.168
Macropores2 *(r ≥ 500 µm)*	0.052	0.019	0.028	0.091	0.019	0.077	0.029

Based on volume in different pore radius classes, the overall performance of the model was satisfying. Only for *L. sativus* statistical indicators given in Fig. 6 demonstrated that the model did not provide an appropriate prediction. In this case the coarse root system induced a shift of r_m,Kosugi_ to higher values. This is contrary to the process described by the convection–dispersion equation underlying the model and therefore could not capture the observed changes. For the other cases volume allocation to different radius classes was predicted satisfactorily. However the corresponding frequency distribution showed an increasing deviation of measured and simulated PSD towards the fine pore classes. This is the result of the diffusion process underlying the physics of the model. The lower boundary condition defines a zero probability flux at r = 0. When pore volume shifts towards the lower boundary, a small volume induces a strong increase in frequency of the fine pore radii near the lower boundary. Measured PSDs on the contrary showed a decreased frequency towards the lower boundary. The volume shift resulted in a distinct peak in volumetric frequency between the upper (pores with r → ∞) and lower boundary (pore of radius r = 0). Such a peak however was not predictable by the model because the diffusion process resulted in an equilibration of frequency over the whole pore range between boundaries.

## Discussion

This study investigated the effect of species with different root systems on field soil pore properties. Over the last decade modern imaging methods have provided new insights into small scale processes at the root-soil interface (e.g. Young and Crawford 2004; Feeney et al. 2006; Moradi et al. 2011). However there is still uncertainty on the importance of roots at higher REV relevant for effective hydraulic processes under field conditions. Furthermore few studies involved sufficient species with variable root characteristics to infer if there were distinctive trends in root effects on soil properties. We used twelve species of different plant families commonly grown as cover crops to investigate modification of macroscopic pore properties in surface near soil where rooting density is highest and most dynamic structural changes occur.

Analysis of root system diversity revealed two dominant rooting types, being (i) a density dominated type with fine axes morphology and (ii) a coarse axes dominated type with lower density. Differentiation in mechanical strength of roots due to axes thickness is essential when studying root impact on soil structure (Jin et al. 2013). Parameters of lognormal root volume distribution according to Scanlan and Hinz (2010) demonstrated that annual herbaceous plants differed in RVD and the standard deviation of distribution. Still they allocated their root volume mainly to fine and very fine axes according to Böhm’s (Böhm 1979) classification. Higher variability might have been obtained by different life forms including shrubs and trees. Also methodological shortcomings have to be considered. Higher differentiation towards very fine roots < 0.2 mm diameter is restricted by the accuracy of root washing and image analysis resolution (Himmelbauer et al. 2004).

The soil pore system was characterized by macroscopic parameters of Kosugi’s lognormal PSD model estimated from infiltration measurements via inverse modeling. Šimůnek et al. (1998) demonstrated that this approach was most appropriate to reproduce effective field hydraulic processes. Furthermore tension infiltrometry covers a comparatively high REV which is an important advantage for representative sampling in the highly variable structural range. Kosugi’s PSD model is often used because of the physical interpretation of its parameters. Hayashi et al. (2006) demonstrated that r_m,Kosugi_ and σ_Kosugi_ were proper indicators for structural porosity. A high r_m,Kosugi_ for a given soil texture class reveals the importance of macroporosity as a product of biotic and abiotic structure forming processes. A narrow pore size distribution (low σ_Kosugi_) with a high frequency of the dominant pore radius class is characteristic for structureless soils. The formation of a secondary, structure related, pore system increases the range of pore radii (high σ_Kosugi_) and shows a more evenly distributed frequency of the single pore classes. Focusing on the shape of the PSD function, we only optimized r_m,Kosugi_ and σ_Kosugi_ while fixing *l* (θ_s_ and K_s_ were taken from measurement and direct evaluation respectively). It is well known that plant roots enhance pore connectivity (e.g. Pagliai and De Nobili 1993; Whalley et al. 2005). However tortuosity is a poorly defined fitting parameter in macroscopic models of hydraulic conductivity (Vervoort and Cattle 2003). Thus it is difficult to define proper initial values and parameter constraints. Furthermore the parameter mostly affected by an inadequate tortuosity value is K_s_, while our study focused on root induced changes in PSD parameters (θ_s_, r_m,Kosugi_ and σ_Kosugi_). Therefore we decided to fix *l* in order to reduce the number of parameters to be estimated and thereby improve the optimization result (Hopmans et al. 2002). Using pore network models might be a way forward to better understand root-pore interactions (Leitner et al. 2013; Hunt et al. 2013).

Feeney et al. (2006) demonstrated that roots effectively micro-engineer the structural arrangement of surrounding rhizosphere soil using 3D imaging. In spite of scale differences up to 5 orders of magnitude (µm to dm) we detected significant root effects on macroscopic pore parameters at the field scale. Particularly total pore volume was substantially increased by RVD when exceeding a lower limit of 0.5 % of pore volume occupied by roots. Several authors demonstrated post-tillage soil settlement to be a main process underlying field pore dynamics (Leij et al. 2002; Schwen et al. 2011; Bodner et al. 2013b). Higher soil porosity in more densely rooted plots was most likely explained by pore stabilization of the loose structure created by pre-seeding chisel tillage. Bodner et al. (2008) had shown pore stabilization in planted compared to bare soil over winter. Also Löfkvist (2005) demonstrated that plant roots could reinforce soil porosity following mechanical subsoil loosening. Species with low rooting density (*F. esculentum, S. cereale*) showed pore loss similar to an unplanted control which was most pronounced in the large macropore range (−33 %) and decreasing to −2 % in the micropore range. *M. officinalis* and *T. alexandrinum* also had low porosity and comparatively low rooting density. However pore loss under this coarse rooted species was not related to macropore degradation, but to changes in microporosity. The exponential relation between RVD and θ_s_ suggests that here RVD was already high enough to avoid macropore loss while strong dominance of coarse axes reduced the fine pore volume.

Horn et al. (1994) and Dexter and Richard (2009) remarked that formation of structural porosity is reflected by two processes, enhanced macroporosity as well as heterogenization of the pore system as a result of finer intra-aggregate pores. Also Milleret et al. (2009) reported that root generated structural pores were found with diameters both smaller and larger than the diameters of penetrating roots. Our results revealed that distinct pore dynamics were induced by coarse rooted and fine rooted species once exceeding a lower limit rooting density. The coarse rooting type of legume species caused a drift towards higher r_m,Kosugi_, shifting pore volume towards the macropore range. The densely rooted species with predominantly fine axes enhanced the heterogenization of the pore system by a dispersion like increase of σ_Kosugi_. Although dense fine rooted species still had 16 % higher macropore volumes compared to unplanted soil and low density *F. esculentum* and *S. cereale*, the coarse rooted legumes increased macroporosity by 30 %. In the dense fine rooted species on the contrary higher σ_Kosugi_ significantly increased micropore volume by 18 %. In the coarse rooted species this pore classes were decreased by 11 %

**Fig. 7:**
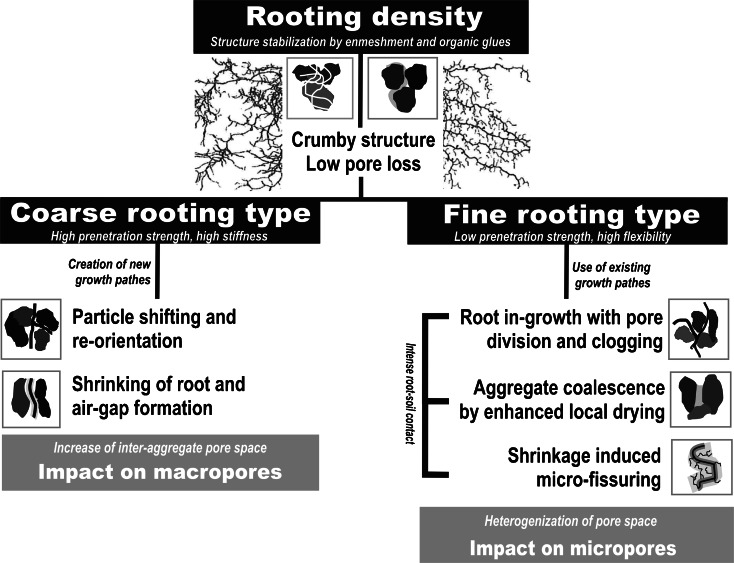
Dominant processes involved in root induced effects on PSD for different rooting types

Figure 7 provides an interpretative framework for these macroscopic pore dynamics, relying on structure forming processes that have been describe at the root-soil interface.

Root and fungal enmeshment constitute the main binding agent at the macro-aggregate level of soil structure (Tisdall and Oades 1982; Miller and Jastrow 1990). Beside this direct effect, also root and fungal exudates are involved in macro-aggregate stabilization. This sustains the common evidence from field soil survey of a crumby, loose structure in densely rooted soil (e.g. Rampazzo and Mentler 2001) and is consistent with the enhanced (macro)porosity of denser rooted soil in our study.

Clark et al. (2003, 2008) pointed to the essential role of root diameter for mechanical root-soil interactions. While the maximum axial force of roots was similar among species, root diameter reduced root buckling when facing mechanical resistance (Clark and Barraclough 1999). At the field scale coarse and tap rooted species were reported to be more effective to alleviate soil compaction (Chen and Weil 2010). In our study axes thickness was strongly related to r_m,Kosugi_. Low frequency of macropores in the PSD indicates that large pores can result from localized structural changes. This is consistent with lower rooting densities of coarse rooted species that still had highest impact in the macropore range.

Several authors described an increase of soil density in a zone of 50–200 μm around roots compared to bulk soil (e.g. Dexter 1987; Dorioz et al. 1993; Whalley et al. 2004). The higher macroporosity under coarse rooted species seems to contradict these findings and points to the challenge of scaling between microscopic and macroscopic phenomena. However, also other studies (e.g. Holtham et al. 2007; Uteau et al. 2013) described an increase in macroporosity by coarse legume roots. Using a pore network model Holtham et al. (2007) suggested that white clover roots caused local structuring of soil, with more pore throats and more throats surrounding large pores compared to the finer roots of ryegrass. This demonstrated how small scale structuring caused major changes in macroscopic processes.

We suggest that coarse roots with high stiffness induced stronger shifting and re-orientation of soil particles upon penetration, counteracting a tight packing between aggregate surfaces and thereby increasing the inter-aggregate void space. Also disruption of large macro-aggregates might result in formation of new inter-aggregate pores (Materechera et al. 1994; Traore et al. 2000; Pierret et al. 2007). Furthermore higher diameter roots may have also created larger air gaps between root and soil in case of drought induced shrinkage (Carminati et al. 2009). Several studies on biopore formation in deeper soil layers, mostly focusing on penetration of compacted or dense zones in the profile, similarly found higher effect of roots with predominantly coarse root diameter (e.g. Williams and Weil 2004; Chen and Weil 2010; Perkons et al. 2014).

Root systems with high density and dominance of fine axes had comparatively lower macropore volumes. Here however the lower macroporosity was not related to a loss in total porosity but to a shift towards fine pore classes. Compared to the coarse systems, large macropores were decreased by 23 %, while fine micropores increased by 74 %. In case of most root volume being allocated to fine axes (low r_m,root_), higher heterogeneity of the pore space (high ς_Kosugi_) was observed. Pore classes that most likely served as preferential growth paths of roots (r > 37.5 µm; Watt et al. 2006; Zobel 2008) were reduced, while micropore volume increased.

We suggest that flexible fine roots could better use existing pore space to penetrate the soil. While stabilizing structure by intense enmeshment, they reduce the macropore space via direct and indirect in-growth effects. Scanlan (2009) considered pore division as an important feature explaining changes in soil hydraulic properties by reduction of pore radius via root in-growth. Dense and fine root systems using existing pore space also provide intense root-soil contact. This enhances local drying and capillary driven particle coalescence (Kirby and Blunden 1991; Kodikara et al. 1999; Cockroft and Olsson 2000; Leij et al. 2002). Indeed there was a strong effect of soil moisture in the fine rooted species. Root induced drying reduces pore radius by coalescence of particles (Ghezzehei and Or 2000). Furthermore drying can lead to formation of macropores (cracks) as well as micropores (fissures) depending on clay content, degree of drying and cyclical drying and re-wetting (Yoshida and Adachi 2001). In our study intermediate clay content of the soil (24 %) and less intense drying during autumn probably limited crack formation. Still the trend to higher microporosity at lower water content that we noticed points to an important role of capillary driven coalescence and micro-fissuring in the depletion zones around roots. The resulting heterogenization of the pore space (high ς_Kosugi_) with substantial increase of microporosity was most evident for species with highest rooting density and volume allocation to fine axes. For the coarser rooted species, the relation between ς_Kosugi_ and root parameters was weak. This indicates that other traits not captured by our sampling method (very fine roots, root hairs, fungal hyphae) or not related to root morphology (e.g. exudation, rhizosphere microbes, abiotic effects) were probably more relevant at the level of micro-aggregation and intra-aggregate porosity (Six et al. 2004).

Within a broader management context, the importance of root induced changes of soil pore properties have been discussed in relation to soil permeability, penetration of compacted layers and enhanced storage porosity. The original concept of soil priming was mainly oriented to improve cash crop root penetration through dense soil (Cresswell and Kirkegaard 1995). Here coarse root systems clearly showed better results due to higher axial strength (e.g. Williams and Weil 2004; Chen and Weil 2010; Perkons et al. 2014). In case of intermediate compaction levels, tap rooted crops with strong root mechanical resistance against buckling (Clark and Barraclough 1999) and perennial forage legumes (Lesturgez et al. 2004) can be sufficiently effective. We consider that inclusion of short growing cover crops in the rotation should be rather considered a precautionary than a curative measure for soil compaction. Particularly for strong compaction or naturally hardset horizons, woody species (Yunusa et al. 2002; Bartens et al. 2008) are be required to effectivly improve penetrability of these layers for subsequent crops. Beyond biopore creation in dense layers, roots can be targeted as a natural management tool for soil structural porosity to enhance water holding capacity as well as saturated hydraulic conductivity. Some crop rotation studies (e.g. Dexter et al., 2001) suggested that roots were directly involved in the improvement of hydraulic behavior at the field scale. Rasse et al. (2000) showed the higher macroporosity and saturated hydraulic conductivity as a result of alfalfa root penetration and the enhanced wet-dry cycles in the rhizosphere. In this context our results show the important contribution of cover crops that influence the aggregation process and thereby influence the formation of a structural pore network. When targeting an aggregation related process, also the biochemical binding agents for aggregate formation and stabilization are essential. Liu et al. (2005) for example showed the causal relation between enhanced aggregate stability and organic carbon input by cover crops. Aggregates underlie a turnover process which is tightly related to the dynamics of their organic binding agents (De Gryze et al. 2006). Therefore plant mediated effects have to be considered was a variable process over time. This is clearly revealed by the results of Głąb et al. (2013) compared long-term effects of different crop rotations They found a significant influence of crop species on water retention. However the crop effect was not stable over time and no long-term rotation effects could be demonstrated.

These results as well as our findings of a root type dependence of hydraulic properties indicate the importance of their dynamic description for hydrological modelling. Green et al. (2003) gave an overview on some empirical approaches that have been used so far. Or et al. (2000) and Leij et al. (2002) were the first in suggesting a physically based model assuming that pore dynamics can be described with a convection–dispersion like equation. This model was developed to simulate post-tillage soil settlement driven by abiotic processes.

Application of the model to root driven pore evolution revealed two problems. First the model could not describe evolution towards a higher r_m,Kosugi_ as observed for the coarse rooted species. Second drift and dispersion shifted a proportion of pore volume to the lower boundary leading to a strong increase in the frequency of very fine pore classes. Although this only slightly affected the predicted pore volume distribution, it revealed that root induced changes were not described appropriately. Root influences were obviously limited to a narrower pore range without affecting very fine textural pore classes. The diffusion process underlying the model tended to an equilibration of pore frequency over the entire radius range and did not reproduce the formation of a distinct peak in the PSD. Model predictions might be improved when defining radius dependent drift and dispersion terms and appropriate boundary conditions to better capture the pore range influenced by roots.

Still it is questionable if a diffusion like process (shift from lower to higher entropy) is adequate to capture the physics of an actively self-organizing biological root-microbe-soil system (Young and Crawford 2004) where energy driven processes lead to a higher order in soil structure. Compared to an abiotic process, formulation of a mechanistic model for pore evolution is more challenging to the higher complexity of a biological system.

## Conclusion

Our study addressed the effect of different root systems on macroscopic pore parameters of the Kosugi PSD model. Characterization of pore properties was done by inverse optimization of tension infiltrometer measurements in a field experiment with twelve cover crops from different plant families. We demonstrated that plant roots essentially conditioned soil pore properties via pore stabilization, macropore formation upon coarse root penetration and pore space heterogenization by dense fine root growth. Pore stabilization was obtained by root systems with a minimum density higher 0.5 % of soil pore volume occupied by plant roots. Comparing coarse and fine root systems with sufficient density to avoid pore loss, distinct structure forming processes were revealed. Formation of macroporosity via a drift of r_m,Kosugi_ to higher values required coarse root systems. We suggested that this was mainly the result of enhanced mechanical resistance of roots against buckling upon soil penetration, leading to shift and re-organization of solid particles in the rhizosphere and consequently a looser packing with more void space. Root systems with high density and strong allocation of their root volume to fine axes can better make use of existing pores as preferential growth paths. They induced a dispersion like change in the PSD via an increase of ς_Kosugi_. This significantly increased micropore volume while reducing the volume of larger pores which were likely used as growth paths. We suggested that aggregate coalescence and micro-fissuring were main causes for the higher microporosity. This is sustained by the influence of soil moisture in addition to root traits.

Our study provides evidence that soil physical quality can be effectively managed by plant roots. Linking the distinct macroscopic changes caused by coarse and fine root systems with new insights into small scale root-pore processes is essential to develop quantitative scaling models and thereby provide appropriate predictive tools for plant based management of soil structure.

## Abbreviations

PSD: Pore size distribution
h_m,Kosugi_: Median pressure head
r_m,Kosugi_: Median pore radius
ς_Kosugi_: Standard deviation of PSD
θ_s_: Saturation water content
*V*: Drift term
*λ*: Dispersivity
RVD: Root volume density
SRL: Specific root length
RD: Root diameter
r_m,root_: Median root radius
REV: Representative elementary volume
